# GelFAP v2.0: an improved platform for Gene functional analysis in *Gastrodia elata*

**DOI:** 10.1186/s12864-023-09260-1

**Published:** 2023-04-04

**Authors:** Jiaotong Yang, Pengfei Li, Yuping Li, Qiaoqiao Xiao

**Affiliations:** Resource Institute for Chinese and Ethnic Materia Medica, Guizhou University of Traditional Chinese Medicine, Guizhou, 550025 China

**Keywords:** *Gastrodia elata*, Functional annotation, Analysis tools, Platform

## Abstract

**Background:**

*Gastrodia elata* (tianma), a well-known medicinal orchid, is widely used to treat various kinds of diseases with its dried tuber. In recent years, new chromosome-level genomes of *G.elata* have been released in succession, which offer an enormous resource pool for understanding gene function. Previously we have constructed GelFAP for gene functional analysis of *G.elata*. As genomes are updated and transcriptome data is accumulated, collection data in GelFAP cannot meet the need of researchers.

**Results:**

Based on new chromosome-level genome and transcriptome data, we constructed co-expression network of *G. elata*, and then we annotated genes by aligning with sequences from NR, TAIR, Uniprot and Swissprot database. GO (Gene Ontology) and KEGG (Kyoto Encylopaedia of Genes and Genomes) annotations were predicted by InterProScan and GhostKOALA software. Gene families were further predicted by iTAK (Plant Transcription factor and Protein kinase Identifier and Classifier), HMMER (hidden Markov models), InParanoid. Finally, we developed an improved platform for gene functional analysis in *G. elata* (GelFAP v2.0) by integrating new genome, transcriptome data and processed functional annotation. Several tools were also introduced to platform including BLAST (Basic Local Alignment Search Tool), GSEA (Gene Set Enrichment Analysis), Heatmap, JBrowse, Motif analysis and Sequence extraction. Based on this platform, we found that the flavonoid biosynthesis might be regulated by transcription factors (TFs) such as MYB, HB and NAC. We also took *C4H* and *GAFP4* as examples to show the usage of our platform.

**Conclusion:**

An improved platform for gene functional analysis in *G. elata* (GelFAP v2.0, www.gzybioinformatics.cn/Gelv2) was constructed, which provides better genome data, more transcriptome resources and more analysis tools. The updated platform might be preferably benefit researchers to carry out gene functional research for their project.

**Supplementary Information:**

The online version contains supplementary material available at 10.1186/s12864-023-09260-1.

## Background

*Gastrodia elata* (*G. elata*) is a typical heterotrophic plant for traditional Chinese medicine, which has been widely used in clinic. It belongs to the genus of *Gastrodia R. Br.* and the family of Orchidaceae with more than 20 synonyms. *G. elata* is mainly distributed in the areas of Asia, including China, Japan, Korea, and India [1]. *G. elata* is a special medicinal plant, its seeds have no endosperm, and its roots and leaves are highly degraded. It cannot absorb nutrients directly from the soil or synthesize required substances through photosynthesis. The growth and development cycle of *G. elata* includes seed, protocorm, juvenile tuber, immature tuber, mature tuber, scape and flower, about 80% of its growth cycle is underground with two fungus *A. mellea* and *Mycena* [2, 3]. *Mycena* offers nutrition for the seed germination of *G. elata*, and *A. mellea* offers nutrition and energy for the vegetative propagation corms of *G. elata* development into tubers [3, 4].

*G. elata* has many pharmacological effects, such as reducing hypertension [5], antioxidant activity [6], antiaging [7], antitumor [8] and immunomodulatory effect [9]. Several ingredients have been identified from *G. elata* including gastrodin, vanillin, vanillyl alcohol, p-endoxybenzyl alcohol, glycoprotein, flavonoid, polysaccharides, etc [10]. Gastrodin is a one of the active component in root of *G. elata*, which has been shown to have a protective effect for neurons hypoxia injury [11]. Polysaccharides extracts from *G. elata* can also attenuate vincristine-evoked neuropathic pain [12]. In addition, *G.elata* is also used as medicine food homology, especially in northwest of China [13]. The dry tuber of *G. elata* has been used for centuries in traditional Chinese medicine, which is considered to be dispels wind, hyperactive liver and dredges collaterals [14]. Otherwise, the Chinese patent medicines with *G. elata* are also widely used in clinic and present positive effects. For example, Tianma Gouteng drink, as a traditional Chinese medicine prescription, has been used clinically to treat cerebral infarction [15]. Banxia Baizhu Tianma decoction is another representative prescription, which has the effect of invigoration the spleen and expectoration phlegm [16]. All these pharmacological effects and functions of *G. elata* cannot be achieved without the active components. Therefore, *G. elata* is a valuable medicinal plant and it is necessary to analyze and explore the key genes regulating the active component accumulation to improve the medicinal value for demand in the future.

With the development of high-through technology, massive data of *G. elata* was accumulated. Since 2018, four genome assemblies of *G. elata* have been released. Sequencing and annotation of *G.elata* genome has been completed by Yuan *et al* in 2018 [2]. Based on *G.elata* genome in 2018, we constructed a basic edition platform for gene function analysis of *G.elata* (GelFAP) [17]. An improved version of *G.elata* genome has been accomplished by Chen *et al* in 2020 [18]. Recently, a high-quality chromosome-level genome sequence of *G.elata* in China has been decoded by Xu et al. [19]. Bae et al. also reported a chromosome level genome of *G. elata* [20]. Improvement and availability of different genomes of *G.elata* can provide an invaluable resource to investigate biosynthesis of its active components. Here, we constructed a new version of gene function analysis platform of *G.elata* based on the chromosome level genome published by Xu et al., which will provide a reference for users to carry out studies on gene function and active component synthesis pathway.

## Materials and methods

### Data resource and functional annotation

Genome data of *G. elata* were derived from National Genomics Data Center (NGDC) (Accession number: GWHBDNU00000000), 45 transcriptome samples in this study were downloaded from Short Read Archive (SRA) database (http://www.ncbi.nlm.nih.gov/sra) and 6 samples was produced by our group (Table S1). GO annotation was collected from Gene Ontology Consortium [21] and KEGG annotation was predicted by GhostKOALA [22]. Sequence of The Ethylene-responsive element binding factor-associated Amphiphilic Repression (EAR) motif-containing proteins and CAZy (Carbohydrate Active Enzyme) proteins were derived from PlantEAR [23] and GAZy database [24] respectively.

### Co-expression network construction

We firstly mapped the transcriptome data to reference genome by hisat2 software [25], TPM (Transcripts Per Million) in each sample was calculated by StringTie software [26]. Secondly, Pearson correlation coefficient (PCC) value between each genes was evaluated by the in house Perl script, we then defined the co-expression network according to the scale free model fit index (R^2^) and nodes number. For the R^2^ less than 0.9, we defined the co-expression network by the best R^2^. For R^2^ more than 0.9, we defined the co-expression network by the largest nodes number. Integration of co-expression network with expression profiles enables effectively analysis of gene functions. Here, differential expressed genes analysis in *G. elata* transcriptome samples was performed and then integrated into the presentation of gene co-expression network.

### Protein-protein interaction (PPI) network construction

As our previous study, rice and maize PPI network were collected from public database RicePPINet [27] and PPIM [28] respectively. To construct *G. elata* PPI network, we also performed orthologous relationship prediction between rice and *G. elata* with a cutoff over 60% bootstrap by InParanoid software [29], as well as maize and *G. elata*. Then we mapped the PPI network in rice and maize to *G. elata*.

### Gene family identification

We firstly used InPranoid software [29] to predicted orthologous relationship of proteins between Arabidopsis and *G.elata*, and further identified CAZy and EAR motif-containing proteins based on orthologous relationship. Using iTAK (Plant Transcription Factor & Protein Kinase Identifier and Classifier) software (http://bioinfo.bti.cornell.edu/cgi-bin/itak/index.cgi) [30], we identified and classified transcription factors and protein kinases in *G.elata*. Based on a hidden Markov model obtained from iUUCD v2.0 (an integrated database of regulators for ubiquitin and ubiquitin-like conjugation, http://iuucd.biocuckoo.org/) [31], ubiquitin families in *G.elata* were identified. Annotation of KEGG pathways for the whole genome were accomplished with GhostKOALA [22]. On the basis of KEGG annotations, CYP450 genes were functionally annotated.

### Construction of GelFAP v2.0

Based on the LAMP (Linux, Apache, MySQL, PHP) technical stack, the platform was constructed. A MySQL database was created by importing all relevant results and data analysis, including gene structure annotation, gene functional annotation, co-expression network, PPI network and gene family classification. Html, PHP, Javascript and CSS languages were used to construct dynamic websites for data presentation and analysis.

### Toolkit for gene function analysis

We introduced gene set enrichment analysis (GSEA) [32] and cis-element enrichment analysis tool as described previously [33, 34]. ViroBlast [35] was used for the construction of Blast analysis. Buels *et al*  developed JBrowse software [36] for the exhibition of omics information, which we also introduced into the platform. We also developed a sequence extraction tool by perl script and induced Heatmap analysis tool by Highchart Javascript.

## Results

### Gene structure and functional annotation

**Fig. 1:**
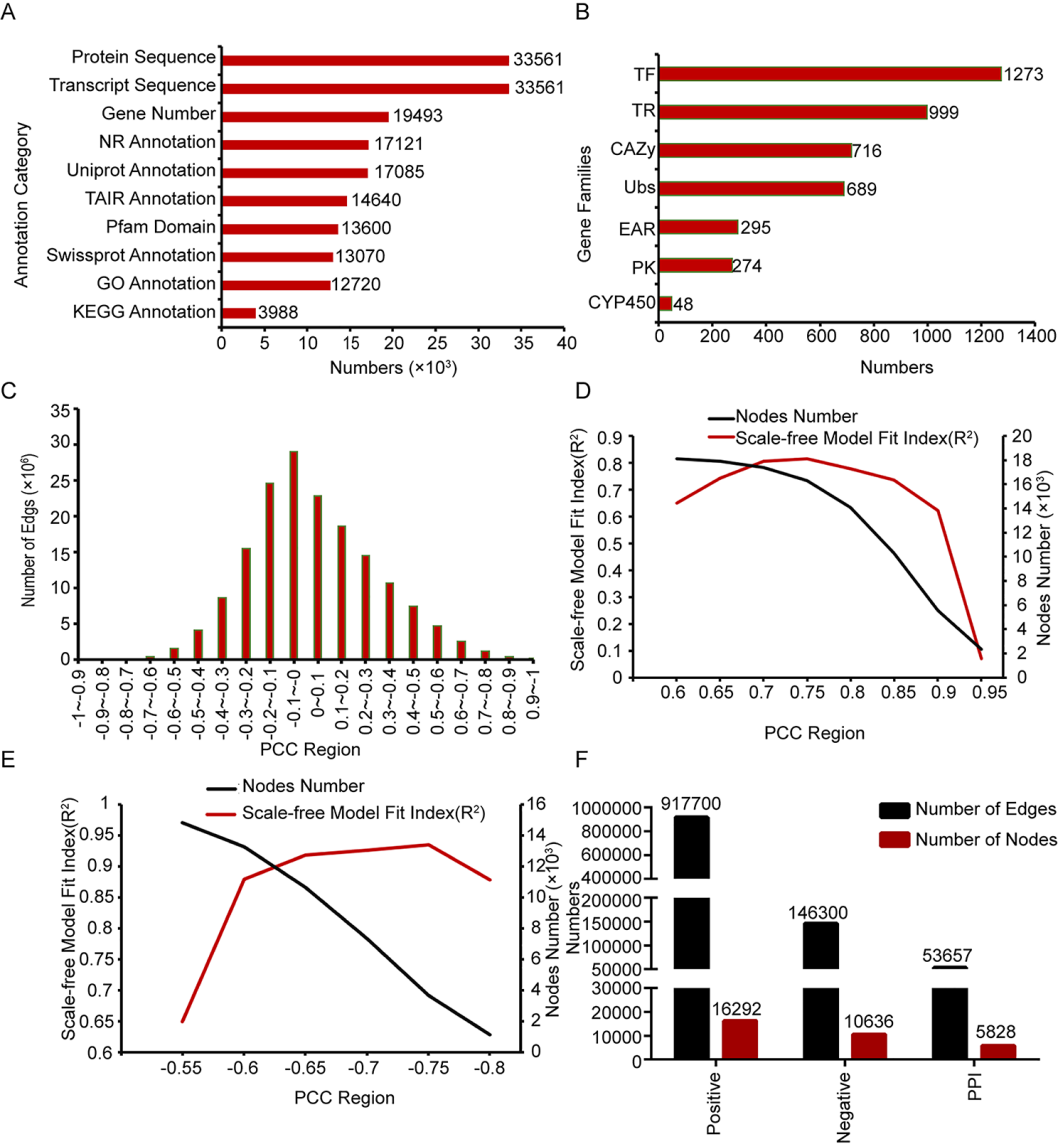
Overview of functional annotation and network construction. (**A**) The number of gene sequences and annotation. (**B**) Gene numbers in different gene families. (**C**) Numbers of gene pairs as PCC changing. (**D**) Nodes number and scale-free model fit (R^2^) distribution as change of PCC. (**E**) Nodes number and scale-free model fit (R^2^) distribution in the negative co-expression network as change of PCC. (**F**) Edges and nodes statistics in the positive, negative co-expression network and PPI network.

We firstly collected genome information of *G.elata* from the NGDC database, including 19,493 genes, 33,561 transcript and 33,561 proteins. By aligning proteins sequence with NR, TAIR, Uniprot and Swissprot database, we annotated 17,121, 14,640, 17,085, 13,070 genes respectively. We also annotated 12,720 genes with GO annotation by InterProScan software [37]. 3988 genes KEGG description was annotated by using GhostKOALA online tools [22] in Kyoto Encyclopedia of Genes and Genomes (KEGG) database (https://www.kegg.jp/) [38–40]. 13,600 genes were subjected to functional annotation of protein domains by the means of the PfamScan software [41] (Fig. 1A).

### Gene family classification

Firstly, iTAK software was used to analyze the transcription factors (TFs), transcription regulators (TRs) and protein kinases (PKs) in *G.elata* and 1273 potential TFs, 999 TRs and 274 PKs were predicted. Secondly, a total of 689 ubiquitin-proteasome coding genes were predicted based on the hidden Markov model (HMM) of the ubiquitin-proteasome downloaded from the iUUCD v2.0 database. Thirdly, All the genes were aligned to the PlantEAR and CAZy database, 716 and 295 genes were assigned to the EAR motif-containing and CAZy families respectively (Fig. 1B).

### Co-expression network

Transcriptome samples from SRA and our group were used to construct co-expression network in *G.elata*. The expression value of each gene was calculated in each sample. We further constructed a expression matrix of genes and calculated the Pearson correlation coefficient (PCC) between each two genes in *G.elata*. PCC algorithm is used to calculate the correlation between every two gene expression, and normalization has no effect on the correlation. The distribution of PCC and gene pairs shown that gene pairs with high correlation are mainly concentrated in middle part (Fig. 1C). By examining the scale-free model fit index (R^2^) for co-expression networks at different cutoff of PCC value, the positive and negative co-expression network were constructed at an appropriate threshold of PCC. The distribution of the highest R^2^ suggested that the PCC > 0.75 was the best threshold for the positive co-expression network (Fig. 1D). We constructed a positive co-expression network with 917,700 edges and 16,292 nodes (Fig. 1F). Different with the positive co-expression network, the scale-free model fit index (R^2^) of negative co-expression network in PCC from − 0.65, -0.7, -0.75 were greater than 0.9, however, the coverage of nodes was the highest when PCC<-0.65 (Fig. 1E). Therefore, PCC less than 0.65 was selected to construct the negative co-expression network. Finally, a negative co-expression network with 146,300 edges and 10,636 nodes was constructed (Fig. 1F).

### Protein-protein interaction (PPI) network

We obtained the rice and maize PPI network from the public database. The PPI network was constructed by mapping the genes in rice and maize to *G.elata* based on orthologous relationship. After removing duplicates of PPI pairs, a total of 53,657 PPI pairs with 5828 nodes was generated (Fig. 1F).

### Construction of GelFAP v2.0

**Fig. 2:**
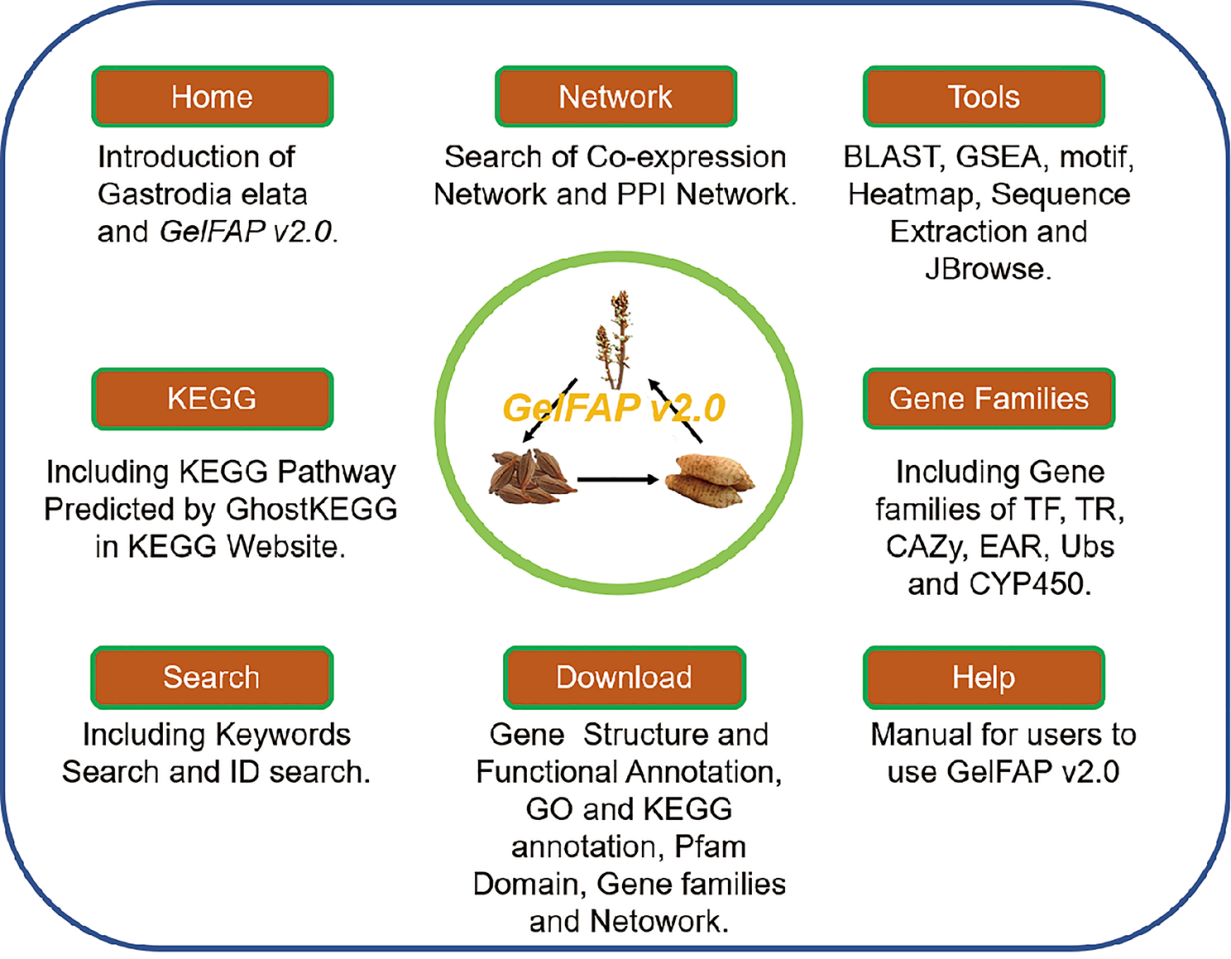
Organizational chart of GelFAP v2.0, including Network, Gene family, Tools, Home, Pathway, Download and Help

An improved platform for gene functional analysis in *G.elata* (GelFAP v2.0) was constructed based on functional annotation, gene family classification, co-expression and PPI network. There are six sections in the framework of GelFAP v2.0, including Home, Network, Pathway, Tools, Gene family, Download and Help. Network section contains PPI and co-expression Network. CYP450, TF, TR, PK, Ubiquitin, GAZy and EAR motif-containing proteins were included in the gene family section. To facilitate gene functional search and analysis of users, seven analysis tools were embedded into GelFAP v2.0, including Search, Blast, Motif Analysis, GSEA, Extract Sequence, Heatmap Analysis and JBrowse. Users could find the genes that they interested in by entering keywords and accurate accession number of gene, transcript or protein in search page. The Blast tool could be used to screen nucleic acid or protein sequences in *G. elata* that are similar to entered sequences. Motif analysis tool was used to search or enrich the motifs in the gene promoter regions. GSEA was used for gene set enrichment analysis, Sequence Extract tool could be used to Extract sequences based on gene accession number and location and Heatmap analysis was used to display gene expression data for candidate gene list. We also integrated JBrowse in GelFAP v2.0 to visualize genomic and transcriptome feature. Download and Help section provided the user with download information as well as user manual for the usage of GelFAP v2.0 (Fig. 2).

### Network display with DEGs in GelFAP v2.0

To integrate gene co-expression/PPI network with expression, the differentially expressed genes (DEGs) were calculated from the three sets of transcriptome data and eight groups of DEGs were finally obtained. Then we constructed joint display node of networks and DEGs. In the display of our network, up-regulated DEGs were marked in red and down-regulated DEGs were marked in blue.

### Functional application

1. Analysis of key enzyme genes in flavonoid biosynthesis pathway.

**Fig. 3:**
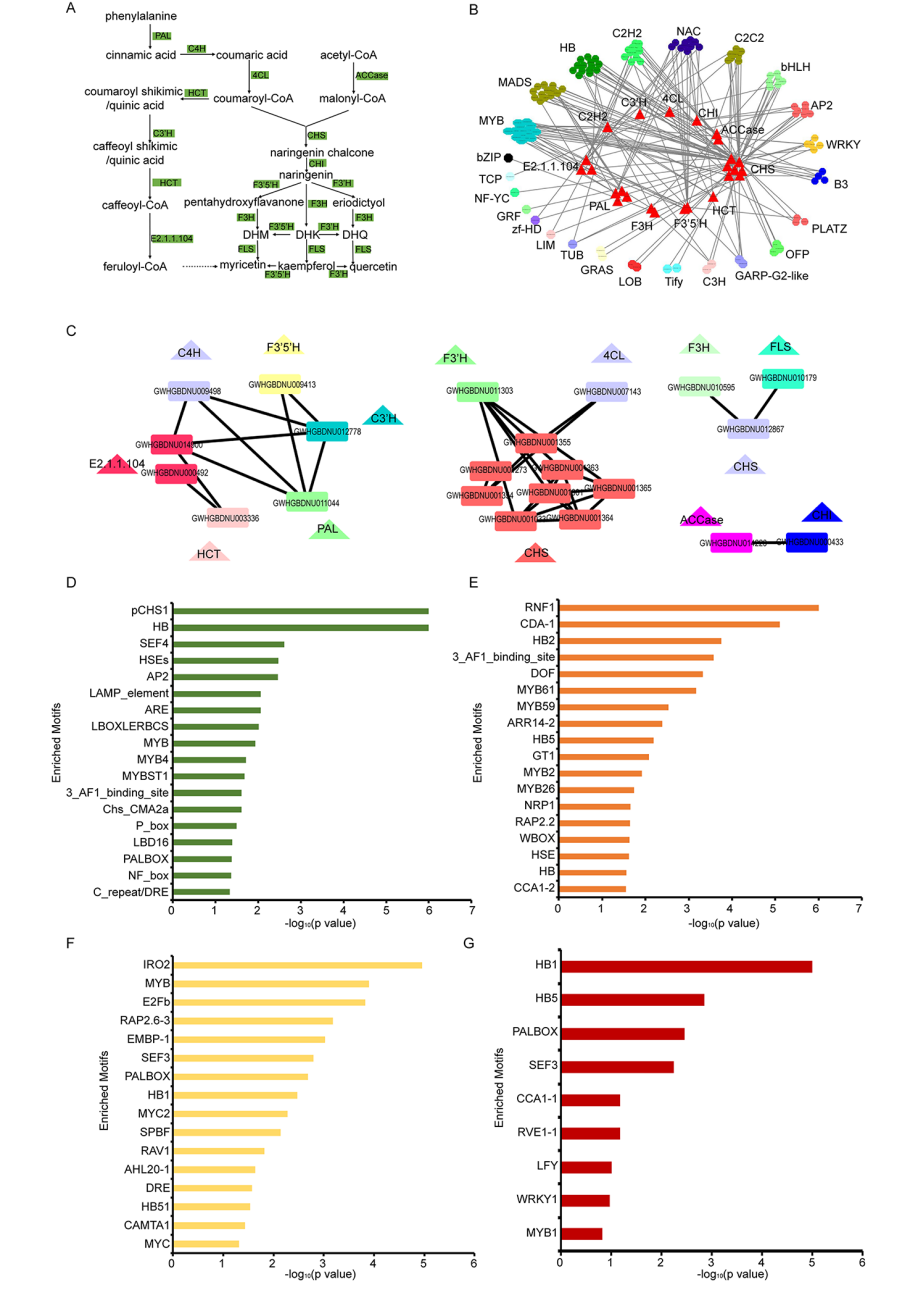
Regulatory analysis of key enzymes in flavonoid biosynthesis pathway in *G. elata*. (**A**) Flavonoid biosynthesis pathway and its key enzyme genes. (**B**) Co-expression relationship between TFs and key enzyme genes in flavonoid biosynthesis. (**C**) Co-expression relationship within key enzymes, which can be divided into 4 modules. (**D**) Motif enrichment analysis results of module1. (**E**) Motif enrichment analysis results of module2. (**F**) Motif enrichment analysis results of module3. (**G**) Motif enrichment analysis results of module4.

Flavonoids are secondary metabolites and play important roles in plant growth and development [42]. Flavonoid biosynthesis is catalyzed by several key enzymes [42], including PAL (phenylalanine ammonia-lyase), C4H (trans-cinnamate 4-monooxygenase), 4CL (4-coumarate–CoA ligase), CHS (chalcone synthase) and so on. The formation of flavonoids has eight different pathways, each leading to the formation of a different type of flavonoid compound [42]. It is reported that flavonoids are both in wild and cultivated *G. elata* [43]. According to KEGG annotation in GelFAP v2.0, there were 43 genes associated to flavonoid biosynthesis pathways were screened (Table S2). Based on the available enzyme information, we found that key enzyme genes mainly formed the backbone of myricetin synthetic pathways (Fig. 3A).

In order to better understand the relationship between key enzyme genes in flavonoids biosynthesis and TFs, co-expression analysis was conducted to identify the TFs which expressions were correlated with the key enzyme genes. The result demonstrated that MYB, HB, NAC and other TFs were co-expressed with these key enzyme genes (Fig. 3B and Table S3). Therefore, key enzyme genes might be regulated by these TFs. We further analyzed the potential co-expression relationships within key enzyme genes in flavonoids biosynthesis, four co-expression relationship modules were found (Fig. 3C and Table S4). Genes in a co-expression module often share similar expression pattern and are potentially regulated by the same TFs. Therefore, motif enrichment analysis of genes in each module were performed using motif analysis tool in our platform. And we found that TFs such as MYB, HB were significantly enriched in genes promoter region in co-expression modules (Fig. 3D, E, F, G). We predicted that co-expression relationship occurred among TFs and target key enzyme genes in flavonoid biosynthesis pathway.

2. Characteristic and functional analysis of *C4H* gene.

**Fig. 4:**
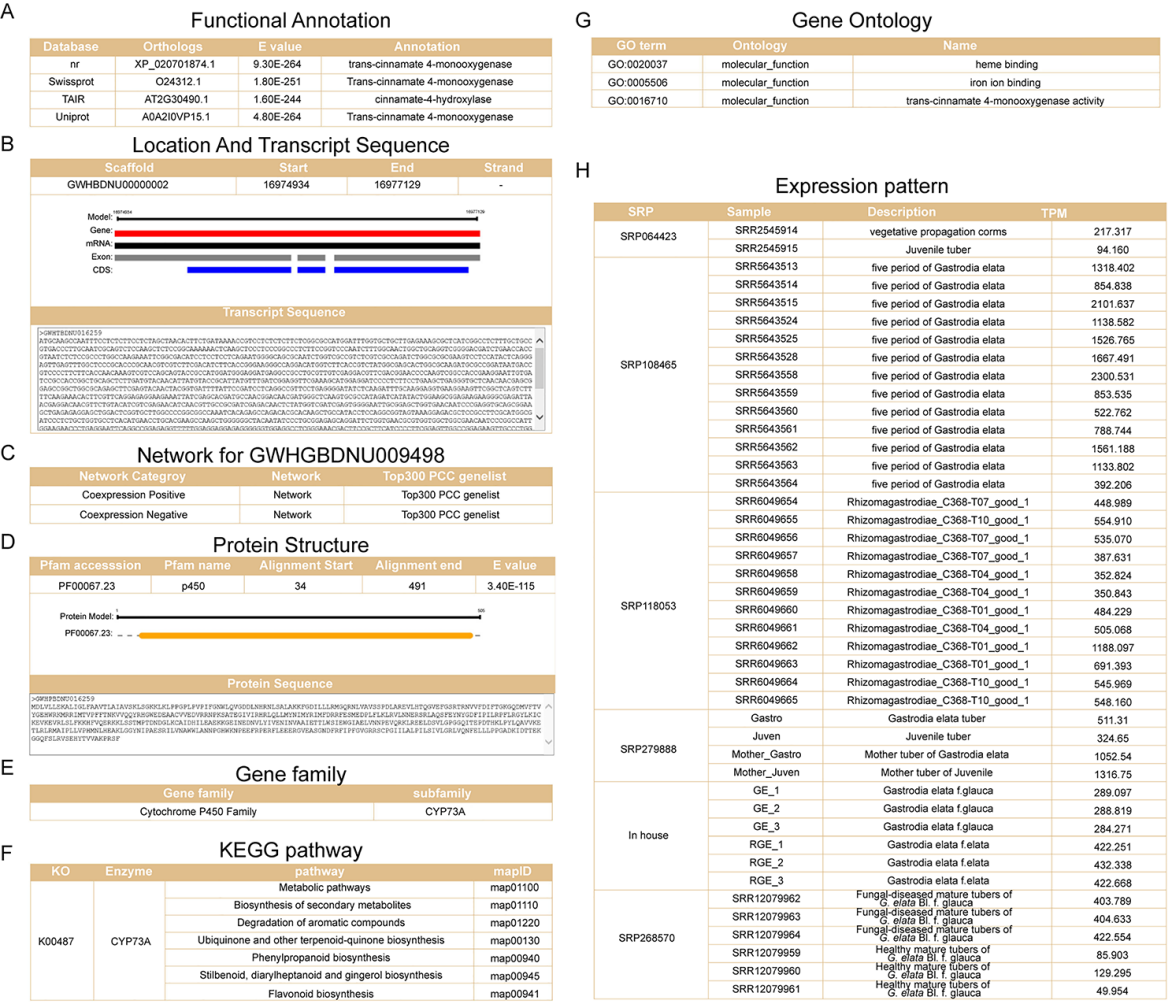
Gene detail page of *C4H* gene. (**A**) Gene functional annotation. (**B**) Location and transcript sequences. (**C**) Network of *C4H*. (**D**) Protein structure and sequence. (**E**) Classification of gene family. (**F**) KEGG annotation. (**G**) GO annotation. (**H**) Expression level in different samples.

**Fig. 5:**
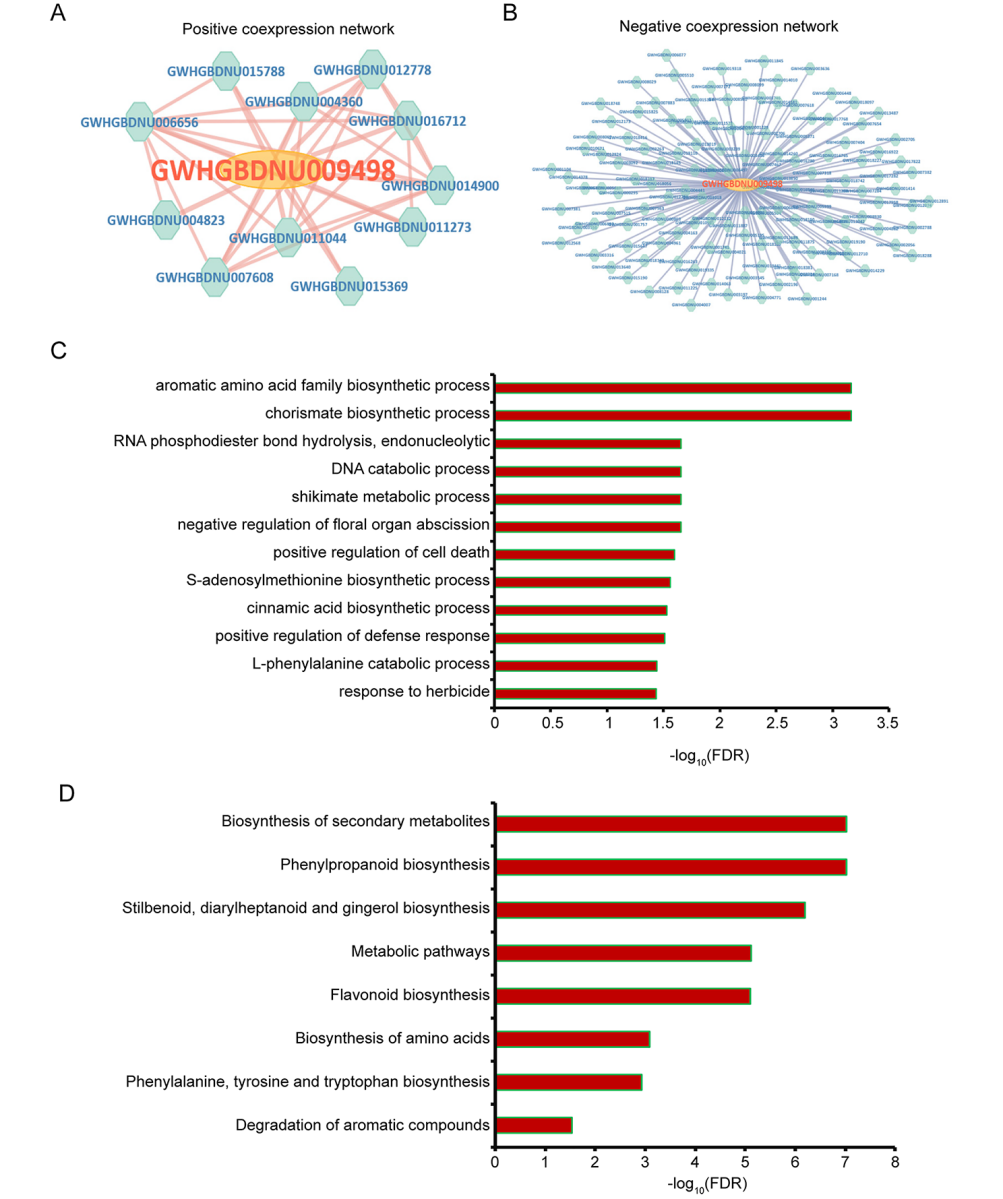
Functional analysis of *C4H* gene. (**A**) Positive co-expression network of *C4H*. (**B**) Negative co-expression network of *C4H*. (**C**) GO enrichment analysis results of *C4H* co-expressed genes. (**D**) KEGG enrichment analysis results of *C4H* co-expressed genes

*C4H* is a key enzyme coding gene that catalyzes the flavonoids biosynthesis [42]. To access the characteristics of *C4H* gene, we utilized functional annotation information, co-expression network and analysis tools in GelFAP v2.0 to perform a comprehensive analysis. Detailed interface of the *C4H* gene provided gene functional annotation (Fig. 4A), transcript location and sequence (Fig. 4B), links for co-expression network (Fig. 4C), protein structure (Fig. 4D), classification for gene families (Fig. 4E), KEGG annotation (Fig. 4F), GO annotation (Fig. 4G) and expression value in different samples (Fig. 4H). Functional annotation, consists of protein functional annotation, KEGG pathway annotation, and GO annotation, provided important information for gene function. KEGG annotation showed the gene involved in flavonoid biosynthesis. In addition, C4H protein contained a single CYP domain and was belong to CYP450 family. Co-expression network analysis suggested that 11 genes positive co-expressed with *C4H* (Fig. 5A) and 133 gene negative co-expressed with *C4H* (Fig. 5B). Next, gene set enrichment analysis (GSEA) was used to determine the enriched GO terms of C4H co-expressed genes. We found that gene sets related to flavonoids biosynthesis were significantly enriched, such as ‘cinnamic acid biosynthetic process’ and ‘L-phenylalanine catabolic process’ (Fig. 5C). GSEA enrichment analysis for KEGG also showed the significantly enriched pathways associated with flavonoids biosynthesis (Fig. 5D).

3. Gene expression analyses for *GAFP4*.

**Fig. 6:**
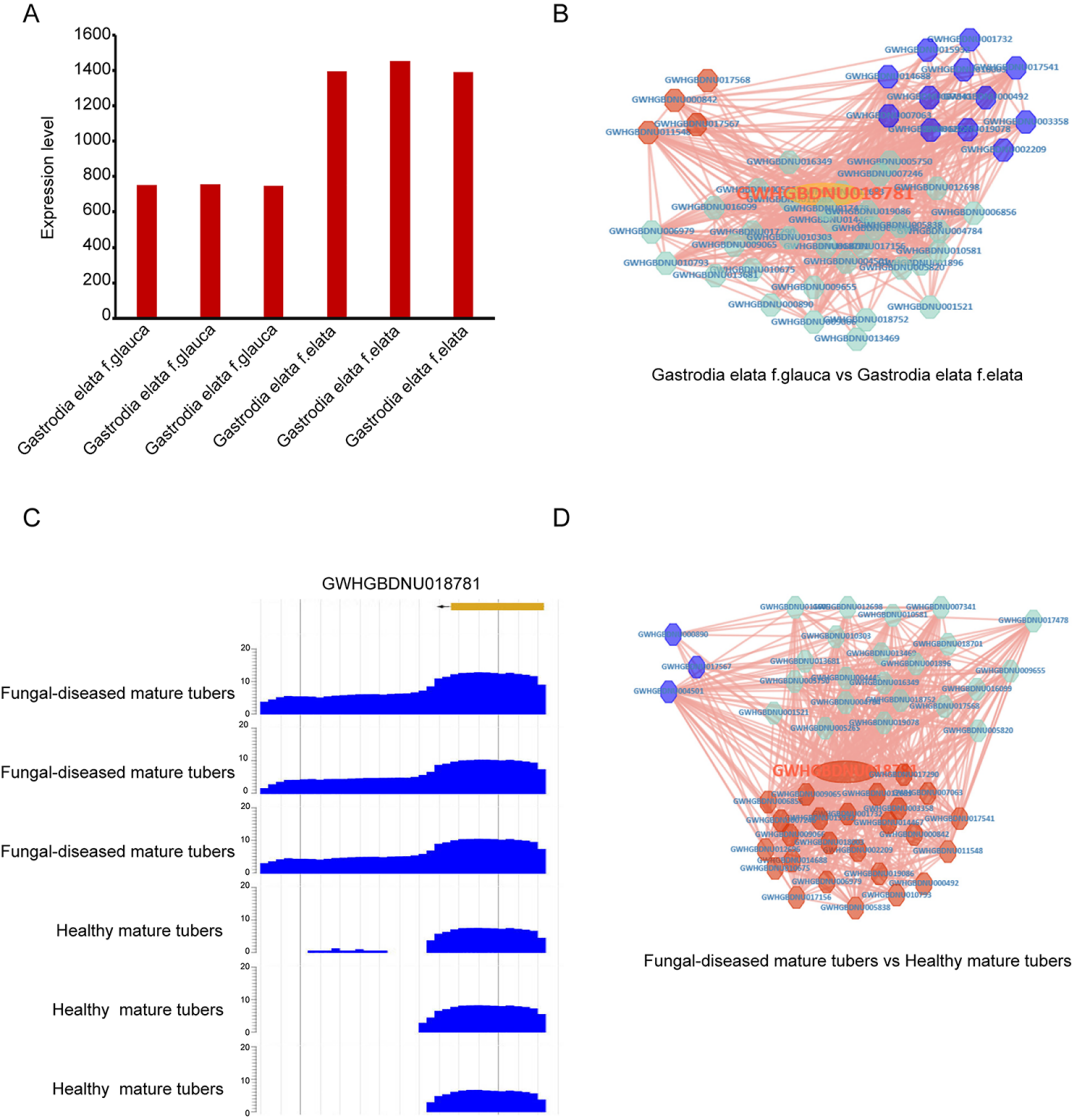
Functional analysis of *GAFP4* gene. (**A**) Expression for *GAFP4* in *G. elata* f.glauca and *G. elata* f.elata. (**B**) The positive co-expression network with DEGs display of *GAFP4* when *G. elata* f.glauca vs. *G. elata* f.elata. (**C**) *GAFP4* expression in fungal-diseased and healthy mature tubers. (**D**) The positive co-expression network with DEGs display of *GAFP4* in fungi-diseased mature tubers vs. healthy mature tubers

*G. elata* usually has a symbiotic relationship with fungi [44, 45], which can cause various diseases. Previous study had shown that *GAFP4* gene had potential antifungal activity [46, 47]. Through the transcriptome analyses, we found that *GAFP4* gene were down-regulated in *G. elata* f.glauca compared to *G. elata* f.elata (Fig. 6A) and its co-expressed genes were also significantly down regulated in *G. elata* f.glauca compared to *G. elata* f.elata (Fig. 6B). The resistance of disease in *G. elata* f.elata was much higher than that in *G. elata* f.glauca [48], which was consistent with *GAFP4* expression. Additionally, we found that the level of *GAFP4* expression was up-regulated by fungi disease (Fig. 6C) and its co-expressed genes were also up-regulated by fungi disease (Fig. 6D). The result was consistent with the *GAFP4* gene function study previously [46, 47].

## Discussion

*G. elata* is an orchid with important biological properties that has a completely mycoheterotrophic lifestyle in nature. There are currently 4 genomes of *G.elata* have been sequenced [2, 18–20], which has provided available resources to study biochemistry, genetics, molecular biology and molecular evolution. Therefore, integration the omics data of *G.elata* is important to assist researchers with scientific research. Finally, we constructed an improved platform for gene function analysis of *G.elata* (GelFAP v2.0) by integrating a new chromosome level genome, transcriptome data, processed annotation data and analysis tools. Compared with the first version of the platform, current version provides better genome data, more transcriptome resources and more analysis tools including Extract Sequence, Heatmap Analysis, JBrowse.

Flavonoids are one of the secondary metabolites in plants and contribute to plant growth and development [42]. They are also widely used in food, medicine and health care. Flavonoids include flavones, flavanols, isoflavones, flavonols, flavanones and flavanonols [42, 49]. For preliminary analysis regulatory mechanism of flavonoid biosynthesis in *G. elata*, we performed gene function and regulatory related analysis by information and tools provided in GelFAP v2.0. Our results showed that MYB, NAC, HB transcription factors might regulate the flavonoid biosynthesis, which has been reported in other related plants [50–54]. For example, expression of key enzyme genes is regulated by MYB–bHLH–WDR complex and further regulated biosynthesis of flavonoids [49]. On the other hand, we used the *C4H* and *GAFP4* gene as examples to introduce the usages of this platform. One PAL, one C3’H and one E2.1.1.104 in flavonoids biosynthesis were directly co-expressed with *C4H* gene (Fig. 3A). One F3’5’H, one E2.1.1.104 and one HCT in flavonoids biosynthesis were indirectly co-expressed with *C4H* (Fig. 3B). Motif enrichment analysis for co-expressed genes also showed enriched TFs such as MYB (Fig. 3C and D). Previously study had suggested that *MYB4* could regulate the expression of the *C4H* gene [55, 56], which encoded a key enzyme in flavonoid biosynthesis. Our analysis may provide references for users to use the platform in the future.

Up until now, many platforms of different plant species have been published to collect and analyze gene function information, such as Rice TOGO Browser [57], ATTED [58], bambooNET [59], NexGenEx-Tom [60], sorghumFDB [61], MCENet [62], croFGD [63], and TeaPVs [64]. Otherwise, several databases contained multiple species for a special plant family, for example, MaGenDB [65] and RPGD [66]. Different platform have different characteristics, most of them incorporated different tools for gene function comparison and analysis to meet different research. In our GelFAP v2.0, we integrated various tools including Search, Blast, Motif, GSEA, Extract Sequence, Heatmap Analysis and JBrowse. At the same time, Network, Gene family, KEGG and Download & Help options were also in the menu bar for researchers to search and download available information. Previously published gene function platforms about plant are mainly contained crops, fruits and vegetables, and few of them was medicinal plants. However, our platform is about medicinal plant *G. elata*, which is rarely found in previous studies, this can provide reference for the subsequent construction of other medical plant gene functional platform. At present, several gene function platforms have not been updated in time, and even some websites cannot be used normally. Our first version GelFAP was constructed in 2020, after that, we continuously paid attention to the research about *G. elata*, timely collected the latest genome and transcriptome data, and constantly updated the information of GelFAP. Thus, GelFAP v2.0 is updated in a short time, which will provide researchers with the latest information for scientific research.

Although we have improved the platform of *G.elata*, it should be pointed out that GelFAP v2.0 also has several limitations and much room to be improved. For example, we only integrated one chromosome genome data in the platform. With the release of different versions of the genome, we will continuously add those latest data in the platform. In the future, we also plan to integrate more new transcriptome data and improve the tools in the platform to meet various requirements for researches in the fields.

We believe that with the continuous development of sequencing technology, cost reduction and long-term investment, *G. elata* multi-omics data will continue to be accumulated. Effective and timely collection and processing of these data and updation of relevant information will be helpful for researchers to carry out their projects. The website is free available at www.gzybioinformatics.cn/Gelv2.

## Electronic supplementary material

Below is the link to the electronic supplementary material.

**Additional file 1: Table S1.** Summary of RNA-seq datasets collected in G.elata. **Table S2.** Key enzymes genes of flavonoid biosynthesis in G.elata. **Table S3.** Co-expression Relationship between key enzymes genes in flavonoid biosynthesis and transcription factor coding genes. **Table S4.** Co-expression Relationship between key enzymes genes in flavonoid biosynthesis.

## Data Availability

Most data analyzed during this study are from the public database. Genome data of G. elata were derived from National Genomics Data Center (NGDC) (Accession number: GWHBDNU00000000) and 45 transcriptome samples in this study were downloaded from Short Read Archive (SRA) database (Accession number: SRP064423, SRP108465, SRP118053, SRP279888, SRP268570). 6 samples were produced by our group which could be downloaded from download page from GelFAP v2.0 (http://www.gzybioinformatics.cn/Gelv2/download&help/download.php).

## References

[1] Lu C, Qu S, Zhong Z, Luo H, Lei SS, Zhong HJ, Su H, Wang Y, Chong CM (2022). The effects of bioactive components from the rhizome of gastrodia elata blume (Tianma) on the characteristics of Parkinson’s disease. Front Pharmacol.

[2] Yuan Y, Jin X, Liu J, Zhao X, Zhou J, Wang X, Wang D, Lai C, Xu W, Huang J (2018). The Gastrodia elata genome provides insights into plant adaptation to heterotrophy. Nat Commun.

[3] Chen L, Wang YC, Qin LY, He HY, Yu XL, Yang MZ, Zhang HB (2019). Dynamics of fungal communities during Gastrodia elata growth. BMC Microbiol.

[4] Kim YI, Chang KJ, Ka KH, Hur H, Hong IP, Shim JO, Lee TS, Lee JY, Lee MW (2006). Seed germination of Gastrodia elata using Symbiotic Fungi, Mycena osmundicola. Mycobiology.

[5] Jiang YH, Zhang P, Tao Y, Liu Y, Cao G, Zhou L, Yang CH (2021). Banxia Baizhu Tianma decoction attenuates obesity-related hypertension. J Ethnopharmacol.

[6] Song E, Chung H, Shim E, Jeong JK, Han BK, Choi HJ, Hwang J (2016). Gastrodia elata Blume Extract modulates antioxidant activity and Ultraviolet A-Irradiated skin aging in human dermal fibroblast cells. J Med Food.

[7] Farooq U, Pan Y, Lin Y, Wang Y, Osada H, Xiang L, Qi J. Structure Characterization and Action Mechanism of an Antiaging New Compound from Gastrodia elata Blume. *Oxid Med Cell Longev* 2019, 2019:5459862. 10.1155/2019/5459862 PMC6526511 31198492

[8] Liu XH, Guo XN, Zhan JP. The effects of Polysaccharide from Gastrodia Elata B1 on cell cycle and caspase proteins activity in H22 tumor bearing mice. Chinese Journal of Gerontology 2015.

[9] Kim NH, Xin MJ, Cha JY, Ji SJ, Kwon SU, Jee HK, Park MR, Park YS, Kim CT, Kim DK (2017). Antitumor and Immunomodulatory Effect of Gastrodia elata on Colon cancer in Vitro and in vivo. Am J Chin Med.

[10] Kho MC, Lee YJ, Cha JD, Choi KM, Kang DG, Lee HS (2014). Gastrodia elata ameliorates high-fructose Diet-Induced lipid metabolism and endothelial dysfunction. Evid Based Complement Alternat Med.

[11] Ng CF, Ko CH, Koon CM, Xian JW, Leung PC, Fung KP, Chan HY, Lau CB (2013). The aqueous extract of Rhizome of Gastrodia elata Protected Drosophila and PC12 cells against Beta-Amyloid-Induced neurotoxicity. Evid Based Complement Alternat Med.

[12] Zhu H, Liu C, Hou J, Long H, Wang B, Guo D, Lei M, Wu W. Gastrodia elata Blume Polysaccharides: A Review of Their Acquisition, Analysis, Modification, and Pharmacological Activities. Molecules 2019, 24(13). 10.3390/molecules24132436 PMC6651794 31269719

[13] Zuo Y, Deng X, Wu Q. Discrimination of Gastrodia elata from Different Geographical Origin for Quality Evaluation Using Newly-Build Near Infrared Spectrum Coupled with Multivariate Analysis. Molecules 2018, 23(5). 10.3390/molecules23051088 PMC6100057 29734695

[14] Hsieh CL, Chiang SY, Cheng KS, Lin YH, Tang NY, Lee CJ, Pon CZ, Hsieh CT (2001). Anticonvulsive and free radical scavenging activities of Gastrodia elata bl. In kainic acid-treated rats. Am J Chin Med.

[15] Tang X, Lu J, Chen H, Zhai L, Zhang Y, Lou H, Wang Y, Sun L, Song B (2021). Underlying mechanism and active ingredients of Tianma Gouteng acting on cerebral infarction as determined via Network Pharmacology Analysis Combined with Experimental Validation. Front Pharmacol.

[16] Xu N, Li M, Wang P, Wang S, Shi H (2022). Spectrum-effect relationship between antioxidant and anti-inflammatory Effects of Banxia Baizhu Tianma Decoction: an identification method of active substances with endothelial cell Protective Effect. Front Pharmacol.

[17] Yang J, Xiao Q, Xu J, Da L, Guo L, Huang L, Liu Y, Xu W, Su Z, Yang S (2020). GelFAP: gene functional analysis platform for Gastrodia elata. Front Plant Sci.

[18] Zhou LK, Zhou Z, Jiang XM, Zheng Y, Chen X, Fu Z, Xiao G, Zhang CY, Zhang LK, Yi Y (2020). Absorbed plant MIR2911 in honeysuckle decoction inhibits SARS-CoV-2 replication and accelerates the negative conversion of infected patients. Cell Discov.

[19] Xu Y, Lei Y, Su Z, Zhao M, Zhang J, Shen G, Wang L, Li J, Qi J, Wu J (2021). A chromosome-scale Gastrodia elata genome and large-scale comparative genomic analysis indicate convergent evolution by gene loss in mycoheterotrophic and parasitic plants. Plant J.

[20] Bae EK, An C, Kang MJ, Lee SA, Lee SJ, Kim KT, Park EJ. Chromosome-level genome assembly of the fully mycoheterotrophic orchid Gastrodia elata. G3 (Bethesda) 2022, 12(3). 10.1093/g3journal/jkab433 PMC8896018 35100375

[21] Gene Ontology C (2015). Gene Ontology Consortium: going forward. Nucleic Acids Res.

[22] Kanehisa M, Sato Y, Morishima K (2016). BlastKOALA and GhostKOALA: KEGG Tools for functional characterization of genome and metagenome sequences. J Mol Biol.

[23] Yang J, Liu Y, Yan H, Tian T, You Q, Zhang L, Xu W, Su Z (2018). PlantEAR: functional analysis platform for plant EAR motif-containing proteins. Front Genet.

[24] Lombard V, Golaconda Ramulu H, Drula E, Coutinho PM, Henrissat B (2014). The carbohydrate-active enzymes database (CAZy) in 2013. Nucleic Acids Res.

[25] Kim D, Paggi JM, Park C, Bennett C, Salzberg SL (2019). Graph-based genome alignment and genotyping with HISAT2 and HISAT-genotype. Nat Biotechnol.

[26] Pertea M, Pertea GM, Antonescu CM, Chang TC, Mendell JT, Salzberg SL (2015). StringTie enables improved reconstruction of a transcriptome from RNA-seq reads. Nat Biotechnol.

[27] Liu S, Liu Y, Zhao J, Cai S, Qian H, Zuo K, Zhao L, Zhang L (2017). A computational interactome for prioritizing genes associated with complex agronomic traits in rice (Oryza sativa). Plant J.

[28] Zhu G, Wu A, Xu XJ, Xiao PP, Lu L, Liu J, Cao Y, Chen L, Wu J, Zhao XM (2016). PPIM: A protein-protein Interaction Database for Maize. Plant Physiol.

[29] Sonnhammer EL, Ostlund G (2015). InParanoid 8: orthology analysis between 273 proteomes, mostly eukaryotic. Nucleic Acids Res.

[30] Zheng Y, Jiao C, Sun H, Rosli HG, Pombo MA, Zhang P, Banf M, Dai X, Martin GB, Giovannoni JJ (2016). iTAK: a program for genome-wide prediction and classification of plant transcription factors, transcriptional regulators, and protein kinases. Mol Plant.

[31] Zhou J, Xu Y, Lin S, Guo Y, Deng W, Zhang Y, Guo A, Xue Y (2018). iUUCD 2.0: an update with rich annotations for ubiquitin and ubiquitin-like conjugations. Nucleic Acids Res.

[32] Yi X, Du Z, Su Z. PlantGSEA: a gene set enrichment analysis toolkit for plant community. Nucleic Acids Res 2013, 41(Web Server issue):W98-103. 10.1093/nar/gkt281 PMC3692080 23632162

[33] Yang J, Yan H, Liu Y, Da L, Xiao Q, Xu W, Su Z (2022). GURFAP: a platform for gene function analysis in Glycyrrhiza Uralensis. Front Genet.

[34] Yu J, Zhang Z, Wei J, Ling Y, Xu W, Su Z (2014). SFGD: a comprehensive platform for mining functional information from soybean transcriptome data and its use in identifying acyl-lipid metabolism pathways. BMC Genomics.

[35] Deng W, Nickle DC, Learn GH, Maust B, Mullins JI (2007). ViroBLAST: a stand-alone BLAST web server for flexible queries of multiple databases and user’s datasets. Bioinformatics.

[36] Buels R, Yao E, Diesh CM, Hayes RD, Munoz-Torres M, Helt G, Goodstein DM, Elsik CG, Lewis SE, Stein L (2016). JBrowse: a dynamic web platform for genome visualization and analysis. Genome Biol.

[37] Jones P, Binns D, Chang HY, Fraser M, Li W, McAnulla C, McWilliam H, Maslen J, Mitchell A, Nuka G (2014). InterProScan 5: genome-scale protein function classification. Bioinformatics.

[38] Kanehisa M, Goto S (2000). KEGG: kyoto encyclopedia of genes and genomes. Nucleic Acids Res.

[39] Kanehisa M (2019). Toward understanding the origin and evolution of cellular organisms. Protein science: a publication of the Protein Society.

[40] Kanehisa M, Furumichi M, Sato Y, Kawashima M, Ishiguro-Watanabe M (2023). KEGG for taxonomy-based analysis of pathways and genomes. Nucleic Acids Res.

[41] El-Gebali S, Mistry J, Bateman A, Eddy SR, Luciani A, Potter SC, Qureshi M, Richardson LJ, Salazar GA, Smart A (2019). The pfam protein families database in 2019. Nucleic Acids Res.

[42] Liu W, Feng Y, Yu S, Fan Z, Li X, Li J, Yin H. The Flavonoid Biosynthesis Network in Plants. Int J Mol Sci 2021, 22(23). 10.3390/ijms222312824 PMC8657439 34884627

[43] Cheng F, Zhang K, Zhao SQ, Zheng J, Fang YX (2009). [The determination of three effective constituents in wild and cultivated Gastrodia elata from Bomi]. Zhong Yao Cai.

[44] Xu JT (1981). [A brief report on the nutrition sources of seed germination of Gastrodia elata (author’s transl)]. Zhong Yao Tong Bao.

[45] Xu JT (1989). [Studies on the life cycle of Gastrodia elata]. Zhongguo Yi Xue Ke Xue Yuan Xue Bao.

[46] Wang Y, Liang C, Wu S, Zhang X, Tang J, Jian G, Jiao G, Li F, Chu C (2016). Significant improvement of Cotton Verticillium Wilt Resistance by manipulating the expression of Gastrodia Antifungal Proteins. Mol Plant.

[47] Wang Y, Liang C, Wu S, Jian G, Zhang X, Zhang H, Tang J, Li J, Jiao G, Li F (2020). Vascular-specific expression of Gastrodia antifungal protein gene significantly enhanced cotton Verticillium wilt resistance. Plant Biotechnol J.

[48] Zhang JQ, Yuan QS, Ouyang Z, Xiao CH, Wei Y, Wang YH, Xu J, Tang X, Wang S, Wang X (2022). [Resistance of different ecotypes of Gastrodia elata to tuber rot]. Zhongguo Zhong Yao Za Zhi.

[49] Xu W, Dubos C, Lepiniec L (2015). Transcriptional control of flavonoid biosynthesis by MYB-bHLH-WDR complexes. Trends Plant Sci.

[50] Yan H, Pei X, Zhang H, Li X, Zhang X, Zhao M, Chiang VL, Sederoff RR, Zhao X. MYB-Mediated Regulation of Anthocyanin Biosynthesis. Int J Mol Sci 2021, 22(6). 10.3390/ijms22063103 PMC8002911 33803587

[51] Song T, Li K, Wu T, Wang Y, Zhang X, Xu X, Yao Y, Han Z (2019). Identification of new regulators through transcriptome analysis that regulate anthocyanin biosynthesis in apple leaves at low temperatures. PLoS ONE.

[52] Dalman K, Wind JJ, Nemesio-Gorriz M, Hammerbacher A, Lunden K, Ezcurra I, Elfstrand M (2017). Overexpression of PaNAC03, a stress induced NAC gene family transcription factor in Norway spruce leads to reduced flavonol biosynthesis and aberrant embryo development. BMC Plant Biol.

[53] Wang J, Lian W, Cao Y, Wang X, Wang G, Qi C, Liu L, Qin S, Yuan X, Li X (2018). Overexpression of BoNAC019, a NAC transcription factor from Brassica oleracea, negatively regulates the dehydration response and anthocyanin biosynthesis in Arabidopsis. Sci Rep.

[54] Dhar MK, Sharma R, Koul A, Kaul S (2015). Development of fruit color in Solanaceae: a story of two biosynthetic pathways. Brief Funct Genomics.

[55] Wang XC, Wu J, Guan ML, Zhao CH, Geng P, Zhao Q (2020). Arabidopsis MYB4 plays dual roles in flavonoid biosynthesis. Plant J.

[56] Zhou M, Sun Z, Wang C, Zhang X, Tang Y, Zhu X, Shao J, Wu Y (2015). Changing a conserved amino acid in R2R3-MYB transcription repressors results in cytoplasmic accumulation and abolishes their repressive activity in Arabidopsis. Plant J.

[57] Nagamura Y, Antonio BA, Sato Y, Miyao A, Namiki N, Yonemaru J, Minami H, Kamatsuki K, Shimura K, Shimizu Y (2011). Rice TOGO browser: a platform to retrieve integrated information on rice functional and applied genomics. Plant Cell Physiol.

[58] Obayashi T, Okegawa T, Sasaki-Sekimoto Y, Shimada H, Masuda T, Asamizu E, Nakamura Y, Shibata D, Tabata S, Takamiya K (2004). Distinctive features of plant organs characterized by global analysis of gene expression in Arabidopsis. DNA research: an international journal for rapid publication of reports on genes and genomes.

[59] Zhao H, Peng Z, Fei B, Li L, Hu T, Gao Z, Jiang Z. BambooGDB: a bamboo genome database with functional annotation and an analysis platform. *Database: the journal of biological databases and curation* 2014, 2014:bau006. 10.1093/database/bau006 PMC3944406 24602877

[60] Bostan H, Chiusano ML (2015). NexGenEx-Tom: a gene expression platform to investigate the functionalities of the tomato genome. BMC Plant Biol.

[61] Tian T, You Q, Zhang L, Yi X, Yan H, Xu W, Su Z. SorghumFDB: sorghum functional genomics database with multidimensional network analysis. *Database: the journal of biological databases and curation* 2016, 2016. 10.1093/database/baw099 PMC4921789 27352859

[62] Tian T, You Q, Yan H, Xu W, Su Z (2018). MCENet: a database for maize conditional co-expression network and network characterization collaborated with multi-dimensional omics levels. J Genet genomics = Yi chuan xue bao.

[63] She J, Yan H, Yang J, Xu W, Su Z (2019). croFGD: Catharanthus roseus Functional Genomics database. Front Genet.

[64] An Y, Zhang X, Jiang S, Zhao J, Zhang F (2022). TeaPVs: a comprehensive genomic variation database for tea plant (Camellia sinensis). BMC Plant Biol.

[65] Wang D, Fan W, Guo X, Wu K, Zhou S, Chen Z, Li D, Wang K, Zhu Y, Zhou Y (2020). MaGenDB: a functional genomics hub for Malvaceae plants. Nucleic Acids Res.

[66] Liu N, Zhang L, Zhou Y, Tu M, Wu Z, Gui D, Ma Y, Wang J, Zhang C (2021). The Rhododendron Plant Genome Database (RPGD): a comprehensive online omics database for Rhododendron. BMC Genomics.

